# Ferroptosis in osteosarcoma: A promising future

**DOI:** 10.3389/fonc.2022.1031779

**Published:** 2022-11-15

**Authors:** Xiang Liu, Shaowen Du, Shengdong Wang, Kaishan Ye

**Affiliations:** ^1^ Department of Orthopedics, Second Hospital of Lanzhou University, Lanzhou, China; ^2^ Key Laboratory of Bone and Joint Diseases of Gansu Province, Second Hospital of Lanzhou University, Lanzhou, China

**Keywords:** ferroptosis, osteosarcoma, molecular mechanisms, tumor treatment, chemotherapy resistance

## Abstract

The incidence of osteosarcoma (OS) is increasing year by year, and the prognosis of patients with advanced OS is extremely poor due to the tendency of recurrence and chemotherapy resistance after surgery. Ferroptosis is a novel form of programmed cell death (PCD) that kills cells through iron-dependent lipid peroxidation. Current studies have shown that ferroptosis is closely related to OS and could reduce chemotherapy resistance to a certain extent, which has great therapeutic potential. In this paper, we review the regulatory mechanism of ferroptosis and its research progress in OS, hoping to provide new help for the clinical treatment of OS.

## 1 Introduction

Osteosarcoma (OS) is a primary musculoskeletal tumor that originates from mesenchymal tissue. It is prevalent in younger age groups and accounts for approximately 2.4% of malignancies in children and adolescents worldwide (1, 2). OS usually affects the metaphysis of long bones, especially the distal femur and proximal tibia. The primary clinical manifestation is persistent localized pain, which may be accompanied by localized masses and limited movement of the adjacent joints (3). OS is highly malignant and prone to chemoresistance and early lung metastases, the prognosis of patients is not optimistic (4). Although surgery combined with multidrug chemotherapy regimens is mainly used to treat OS, the 5-year survival rate of OS remains unsatisfactory due to drug resistance problems (5). As the clinical treatment of OS has reached a “bottleneck” stage, it is essential to develop new and effective treatment strategies.

Ferroptosis is a novel form of programmed cell death (PCD), officially named by Dixon et al. in 2012 (6). Ferroptosis is closely associated with iron overload, reactive oxygen species (ROS) accumulation, and lipid peroxidation reactions. Iron dependence is the main feature. The processes of ferroptosis include: (a) intracellular iron overload leading to massive accumulation of ROS, (b) disruption of redox homeostasis, (c) lethal lipid peroxidation reactions, and cell death (6). The mechanisms of ferroptosis are complex and involve multiple metabolic processes, including amino acid metabolism, iron metabolism, and lipid metabolism (7). As a current research hotspot, ferroptosis has been shown to be therapeutic for a variety of tumors and is expected to resolve chemotherapy resistance caused by traditional pro-apoptotic pathways (8, 9). Herein, we provide a detailed description of the mechanisms of ferroptosis and the therapeutic role of ferroptosis regulation in OS. We hope that our review can provide some references for further research.

## 2 Overview of ferroptosis

### 2.1 Discovery and features of ferroptosis

Before ferroptosis was formally proposed, Dolma et al. (10) identified a synthetic compound, erastin, that can kill cells in a non-apoptotic pathway. On this basis, Dixon et al. (6) further investigated in 2012 and found that the cytotoxic effect of erastin differs from other programmed cell death, such as apoptosis, necroptosis, and autophagy. Its occurrence depends on the mediation of the metal element iron and presents unique morphological changes under the microscope, including a decrease in mitochondrial volume, an increase in membrane density, and a decrease or loss of cristae, while the structure of the nucleus is intact. Then, they named this particular form of cell death ferroptosis. In addition to iron dependency, ferroptosis has been shown to be closely associated with lipid peroxidation due to the accumulation of intracellular ROS (11). Other inhibitors of programmed cell death do not inhibit the progression of ferroptosis, whereas antioxidants and iron chelators achieve inhibitory effects (12).

### 2.2 Regulatory mechanism of ferroptosis

**Figure 1:**
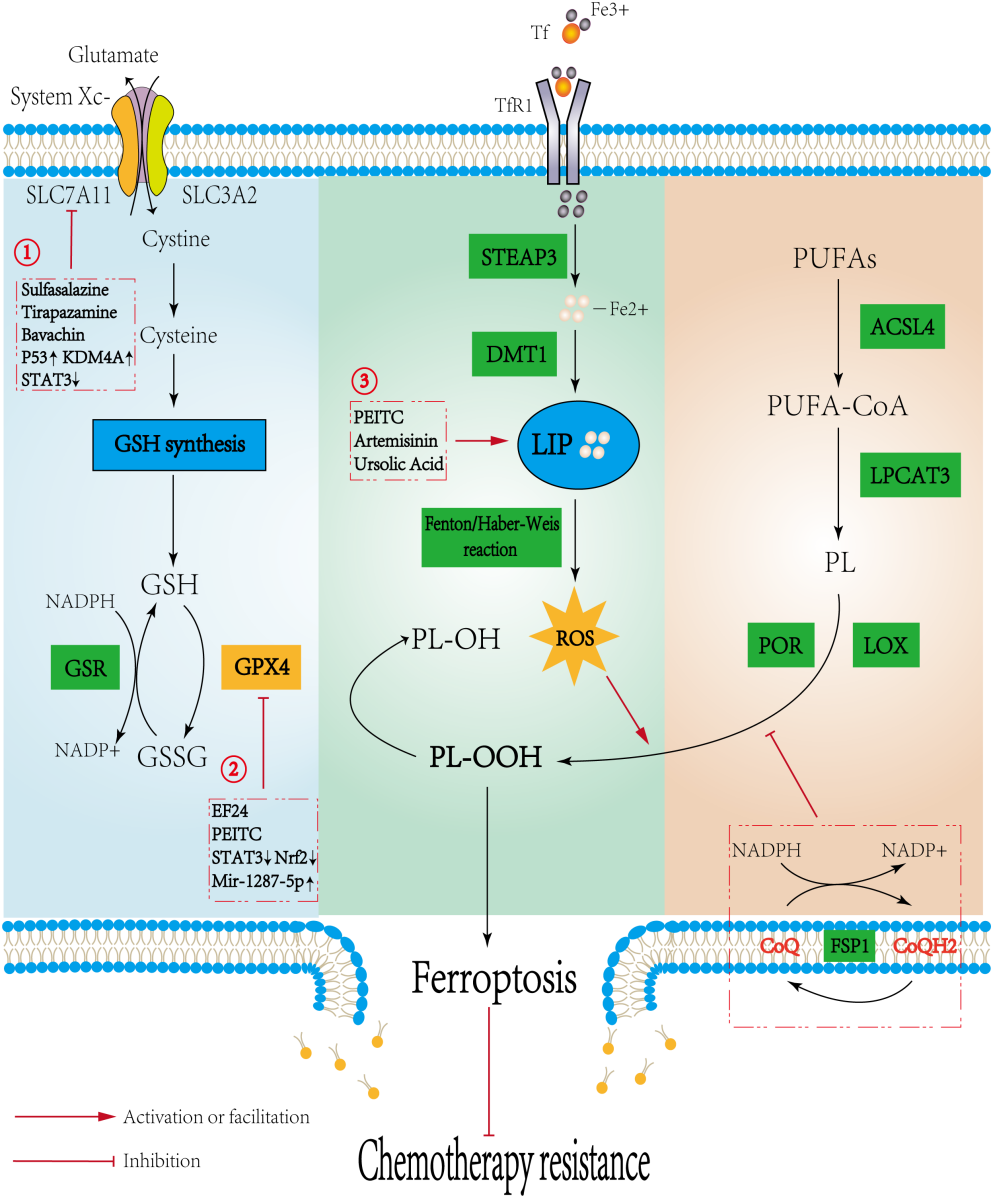
Mechanisms regulating ferroptosis and the potential role in osteosarcoma. Three pathways initiate the process of ferroptosis: the canonical GSH-GPX4 pathway, iron metabolism pathway, and lipid metabolism pathway. The Potential mechanisms of ferroptosis in osteosarcoma are as follows: 1. Sulfasalazine, Tirapazamine, and Upregulating P53 or KDM4A could promote ferroptosis by directly inhibiting the activity of SLC7A11. Bavachin indirectly reduces SLC7A11 expression by downregulating STAT3 to upregulate P53 expression. 2. EF24 and PEITC, upregulating Mir-1287-5p and inhibiting STAT3/Nrf2 signaling pathway could inhibit GPX4 to induce ferroptosis; 3. PEITC, Artemisinin, and Ursolic acid induce ferroptosis by increasing Fe^2+^ within the LIP. Promoting ferroptosis reduces chemotherapy resistance.

Yang et al. (13) first identified the ability of glutathione peroxidase 4 (GPX4) to inhibit the cytotoxic effects of various ferroptosis inducers under the synergistic effect of glutathione (GSH) and established the classical regulatory axis of ferroptosis with GSH-GPX4 as the core. Since then, an increasing number of studies have begun to focus on the exploration of new mechanisms. The main pathways currently regulating ferroptosis include the GSH-GPX4 pathway, iron metabolism pathway, and lipid peroxidation pathway (**Figure 1**). In addition, dihydroorotate dehydrogenase (DHODH), voltage-dependent anion channels, and the GTP cyclohydrolase 1 (GCH1)-tetrahydrobiopterin (BH_4_) have also been found to affect the process of ferroptosis.

#### 2.2.1 GSH-GPX4 regulating axis

The GSH-GPX4 axis is considered to be the classic regulatory axis of ferroptosis, and a large number of studies on ferroptosis are currently focused on it. GPX4 could reduce cytotoxic lipid peroxides to nontoxic lipid alcohols, interrupt the lipid peroxidation chain reaction, and thus inhibit ferroptosis (14). VUČKOVIĆ et al. (15) found that both GPX4 knockdown and the GPX4 inhibitor RSL3 could induce ferroptosis. GSH is an important antioxidant in mammals and is available in both reduced (GSH) and oxidized (GSSG) forms (16). GSH can provide electrons for the reduction of lipid peroxides by GPX4, insufficient GSH level leads to a decrease in GPX4 efficacy, and the accumulation of lipid peroxides causes ferroptosis (17). System Xc^-^ is involved in the regulation of ferroptosis through the GSH-GPX4 axis by controlling the synthesis of GSH. System Xc^-^ is a heterodimer composed of a glycosylated heavy chain SLC3A2 and a non-glycosylated light chain SLC7A11. When its activity is inhibited, the uptake and transformation of cystine and cysteine are reduced, and then the balance between intracellular GSH synthesis and consumption is disrupted, ultimately inducing ferroptosis (18). The activity of System Xc^-^ was positively correlated with the expression level of its light chain SLC7A11 (19). Recent studies found that activation of the P53 gene could exert its tumor suppressor effect by downregulating the expression of SLC7A11 to inhibit System Xc^-^ (20, 21). Notably, the uptake and transformation of cystine and cysteine are dependent on glutamine and NADPH, while NADPH is derived from the pentose phosphate pathway of glucose (22). This finding indicated that SLC7A11 made tumor cells highly dependent on glucose and glutamine. Several studies had confirmed that when GULT or glutaminase inhibitors were used on tumor cells with high expression of SLC7A11, the mortality of tumor cells was significantly increased, and this effect was independent of the GSH-GPX4 axis (23–25). These results suggested that the nutrient dependence caused by SLC7A11 may cooperate with ferroptosis to achieve better anticancer effects. Therefore, direct inhibition of GPX4 or indirect inhibition of GPX4 reducing ability by decreasing GSH synthesis could induce ferroptosis, which provides more possibilities for targeting the GSH-GPX4 axis.

#### 2.2.2 Iron metabolism pathway

Ferroptosis, as the name suggests, is closely related to iron metabolism. Iron is involved in the synthesis of several important proteases in the organism and is an essential and basic element in the vital activities of the human body (26). Fe^3+^ is usually absorbed into the blood by the small intestine and transported through cell membranes by transferrin (Tf). Subsequently, it is endocytosed into cells under the mediation of transferrin receptor 1 (TfR1). Intracellular Fe^3+^ is reduced to Fe^2+^ by the six-transmembrane epithelial antigen of the prostate 3 (STEAP3). A portion of Fe^2+^ is transported to the outside of the cell by ferroportin (Fpn), while the rest is transferred to ferritin (Fn) and the labile iron pool (LIP) in the cytoplasm by divalent metal transporter 1 (DMT1) (27). Under pathological conditions, large amounts of Fe^2+^ accumulate in the LIP and generate large amounts of hydroxyl radicals through the Fenton and Haber-Weiss reactions, which cause a sudden rise in ROS, trigger lipid peroxidation and eventually induce ferroptosis (28). Therefore, intracellular iron overload caused by abnormal iron metabolism is one of the necessary conditions for the occurrence of ferroptosis. A variety of genes and proteins involved in maintaining iron homeostasis have been shown to play important roles in regulating the sensitivity of cells to ferroptosis (29). In addition, compared with normal cells, tumor cells have a greater demand for iron due to their hyperproliferative capacity, and the content of intracellular iron is also relatively increased (30). This indicates that tumor cells may be more sensitive to iron, and targeting iron metabolism may be an effective strategy to induce ferroptosis in tumor cells.

#### 2.2.3 Lipid peroxidation pathway

Lipid peroxidation is the last event before cell death *via* ferroptosis. Polyunsaturated fatty acids (PUFAs) involved in the synthesis of membrane phospholipids are easily oxidized by ROS and produce a large number of lipid peroxides. Lipid peroxides and their by-products disrupt the normal structure and function of membranes, leading to the occurrence of ferroptosis (31, 32). Therefore, the content of PUFAs in membrane phospholipids was positively correlated with the sensitivity of cells to ferroptosis. Recent studies have shown that acyl-CoA synthetase long-chain family member 4 (ACSL4) and lysophosphatidylcholine acyltransferase 3 (LPCAT3) were upregulated in ferroptotic cells. The binding process of coenzyme A (CoA) to PUFAs depends on the catalysis of ACSL4, and the products form membrane phospholipids under the esterification of LPCAT3. ACSL4 and LPCAT3 synergistically increased the content of PUFAs in membrane phospholipids, making the membrane more susceptible to peroxidation in the presence of ROS and leading to ferroptosis (33, 34). Inhibition of ACSL4 or LPCAT3 by genetic or pharmacological suppression could act as a specific anti-ferroptosis pathway (35). In addition, Lei et al. (36) demonstrated that ionizing radiation induced ferroptosis by activating ACSL4. Notably, several studies have shown that LPCAT3 could affect the tumor microenvironment (TME). Knockdown of LPCAT3 not only inhibited ferroptosis but also contributed to the production of lipid-rich tumor-associated macrophages (TMEs), which impede host anticancer immunity and maintain the survival of cancer cells (37, 38). Ferroptosis suppressor protein 1 (FSP1) is a ferroptosis regulator parallel to GPX4, that can still inhibit ferroptosis in the case of GPX4 inactivation (39). BERSUKER et al. (40) found that the inhibitory effect of FSP1 was mainly through the continuous reduction of oxidized coenzyme Q10 (CoQ10) in cells, enhancing its ROS capture ability, and thereby inhibiting ferroptosis. In addition, lipoxygenase (LOX) and cytochrome P450 oxidoreductase (POR) have been shown to play important roles in lipid peroxidation reactions. LOX is a class of nonheme iron-containing enzymes that are directly involved in the esterification process of PUFAs. The overexpression of LOX-5, LOX-12, and LOX-15 could increase the susceptibility of cells to ferroptosis, and LOX inhibitors have also been shown to have potent anti-lipid peroxidation effects (41). POR is an electron donor that provides electrons during cytochrome P450-catalyzed lipid peroxidation (42). Zou et al. (43) found that POR was closely related to ferroptosis in tumor cells through genome-wide screening, and downregulation of POR expression could significantly inhibit lipid peroxidation, thereby exerting anti-ferroptosis effects in a variety of tumor cells.

#### 2.2.4 Other pathways

Except for FSP1, DHODH and GCH1-BH4 were also found to inhibit ferroptosis in a GPX4-independent way. GCH1, which generates the endogenous metabolite BH4, was found to be an inhibitor of ferroptosis in a CRISPR activation screen and an enhancer of ferroptosis in a CRISPR loss-of-function screen (44, 45). GCH1 inhibits ferroptosis by a two-pronged mechanism: (a) GCH1 produces the lipophilic antioxidant BH4, which functions similarly to CoQ10 to prevent lipid peroxidation, (b) GCH1 reduces the content of PUFAs in membrane phospholipids to enhance resistance to ferroptosis. DHODH inhibits lipid peroxidation by increasing COQ10. Specifically, DHODH mainly acts in mitochondria (46). Mitochondria are the primary producers of ROS and play an important role in oxidative stress (47). It was shown that the voltage-dependent anion channel protein 2/3 (VDAC2/3) on the outer mitochondrial membrane opened under the induction of erastin, leading to iron overload in the mitochondria. However, the detailed mechanism of VDAC2/3 and ferroptosis is still being explored (48).

## 3 Research progress on ferroptosis in osteosarcoma

### 3.1 Ferroptosis inhibits osteosarcoma progression

A variety of drugs and molecules have been shown to effectively inhibit the progression of OS by inducing ferroptosis (**Table 1**). Sulfasalazine is commonly used in the treatment of rheumatoid arthritis and inflammatory bowel disease. Sulfasalazine plays a role in promoting ferroptosis in various tumors by inhibiting SLC7A11 to reduce the synthesis of GSH (59). Liu et al. (49) found that sulfasalazine could effectively induce ferroptosis in mouse OS cells, and the effect was more significant when combined with iron overload. Tirapazamine (TPZ) is an anticancer drug targeting hypoxic tumor cells. It achieves anticancer effects by increasing intracellular ROS, and its efficacy is enhanced when combined with cisplatin (60). Shi et al. (53) demonstrated that TPZ had a strong inhibitory effect on OS cells. Similar to sulfasalazine, downregulation of SLC7A11 to induce ferroptosis was one of the mechanisms by which it exerted anticancer effects.

**Table 1:** Interventions and reagents targeting ferroptosis for osteosarcoma.

Intervention methods or reagents	Osteosarcoma cell lines	Mechanism	Effects on cells	Reference
Sulfasalazine	K7M2	Inhibiting SLC7A11	Inducing ferroptosis	(49)
miR-1287-5p	U2os, Saos-2	Inhibiting GPX4	Inducing ferroptosis	(50)
Ursolic Acid	HOS, 143B	Degrading ferritin by activating autophagy and inducing intracellular overload of Fe^2+^	Inducing ferroptosis and reducing drug resistance effect of cisplatin	(51)
Bavachin	MG63, HOS	Inhibiting SLC7A11 by impairing STAT3 and enhancing P53	Inducing ferroptosis	(52)
Tirapazamine	HOS, 143B, U2os	Inhibiting SLC7A11	Inducing ferroptosis	(53)
KDM4A	143B, HOS	Promoting SLC7A11 transcription by inducing H3K9me3 demethylation	Inhibiting ferroptosis	(54)
EF24	U2os, Saos-2	Upregulating HMOX1 to suppress GPX4 expression	Inducing ferroptosis	(55)
PEITC	143B, HOS, U2os, K7M2, MG63	Altering iron metabolism, disturbing the Redox Balance, and activating the ROS-related MAPK signaling pathway	Simultaneously inducing ferroptosis, apoptosis, and autophagy	(56, 57)
STAT3 inhibitor	Saos-2, MG63	Inhibiting STAT3/Nrf2/GPX4 signaling pathway	Inducing ferroptosis and increasing sensitivity to cisplatin	(58)

The extracts of some natural plants or traditional Chinese medicines have the advantages of good anticancer effects, low toxicity, and few side effects, and have good therapeutic effects in various tumors. Phenethyl isothiocyanate (PEITC) is abundant in cruciferous vegetables and has good anticancer effects in ovarian, colon, breast, and oral mucosa cancers (61). Lv et al. (56, 57) showed that PEITC could not only regulate the expression of TfR1 and Fn but also directly inhibit the activity of GPX4, which induced ferroptosis *in vivo* and *in vitro* through dual pathways. In addition to ferroptosis, PEITC also induced apoptosis and autophagy in OS cells. Lin et al. (55) found that the cytotoxic effect of the curcumin synthetic analog EF24 on OS cells could only be reversed by ferroptosis inhibitors, while other cell death pathway inhibitors were ineffective. The above two experimental results show that ferroptosis could coexist with other forms of programmed cell death, induction of ferroptosis alone or in combination with other forms of programmed cell death could achieve good anticancer effects. Luo et al. (52) found that Bavachin, an extract of traditional Chinese medicine psoralea, could upregulate the expression of P53 by inhibiting the activity of signal transducer and activator of transcription 3 (STAT3), which in turn inhibited the activity of SLC7A11 and promoted ROS to induce ferroptosis. Artemisinin is the first-line drug for the clinical treatment of malaria. Studies have shown that artemisinin can induce ferroptosis in various tumor cells (62). Isani et al. (63) found that OS cells killed by artemisinin have morphological characteristics similar to those of ferroptosis cells, and the iron content in the cytoplasm was significantly abnormal, but the specific mechanism is still unclear.

In addition, microRNA-1287-5p was also found to inhibit OS through ferroptosis. Xu et al. (50) proved that microRNA-1287-5p could induce ferroptosis in OS cells by directly inhibiting GPX4.

### 3.2 Ferroptosis reduces chemotherapy resistance in osteosarcoma

Chemotherapy resistance is the main reason accounting for the poor prognosis of many cancers, including OS. Chemotherapy resistance is related to various mechanisms, among which ROS-mediated disruption of redox homeostasis is one of the key factors leading to it. Tumor cells can enhance their tolerance to oxidative stress by inhibiting ROS production and develop chemotherapy resistance (64). Ferroptosis causes ROS overload and disrupts redox homeostasis, which is the main reason for reducing chemotherapy resistance (65). Studies have shown that induction of ferroptosis can reduce the resistance of multidrug-resistant tumor cells to chemotherapeutic drugs to a certain extent (66).

Liu et al. (58) screened drug-resistant OS cells in a certain concentration of cisplatin first and then conducted a series of comparative experiments on drug-resistant and nonresistant cells. They found that the level of GPX4 was significantly higher in drug-resistant cells. Under the same cisplatin concentration as the screening concentration, the mortality of drug-resistant cells treated with ferroptosis inducers was significantly increased, while the mortality of nonresistant cells treated with ferroptosis inhibitors was significantly decreased. Chen et al. (54) found that the histone demethylase KDM4A reduced the sensitivity of OS cells to cisplatin by repressing the transcription of SLC7A11 to inhibit ferroptosis. In contrast, inhibition of KDM4A promoted ferroptosis and enhanced the efficacy of cisplatin. Subsequently, by establishing a mouse model, they found that the induction of ferroptosis not only reduced the size of the tumor but also inhibited the lung metastasis of OS. Ursolic acid, an anticancer active substance extracted from kiwi fruit, can inhibit tumor progression, induce tumor cell differentiation and inhibit angiogenesis (67). A recent study found that ursolic acid could activate the autophagic process of ferritin, increased the level of iron in cells, and reduced the resistance of OS cells to cisplatin in a manner that induced ferroptosis (51). Fu et al. (68) constructed a new type of nanomedicine, that integrated ferrate and doxorubicin into nanocarriers, directly increased the intracellular iron content to induce ferroptosis through the exogenous pathway. The expression of multidrug resistance gene (MDR) and P-glycoprotein (P-gp) in OS cells could be downregulated by this nanomedicine treatment, thereby increasing the sensitivity to doxorubicin.

### 3.3 Associated predictors of ferroptosis in osteosarcoma

A number of studies have analyzed differentially expressed genes and long non-coding RNAs (lncRNAs) during ferroptosis in OS through bioinformatics, and finally identified 26 genes associated with OS prognosis (69–73) (CBS, EGFR, COCS1, PTN, PGD, ZEP36, DLL1, EOMES, ERCC2, G6PD, MYC, SLC39A8, etc.) and 8 lncRNAs (74, 75) (RPARP-AS1, PARD6G-AS1, GAS5, UNC5B -AS1, LINC01060, AC124798.1, AC090559.1, and AC104825.1), which created a new prognostic model for OS patients. In-depth analysis of ferroptosis genes related to OS prognosis through a large database can not only provide directions for basic research but also provide more possibilities for subsequent clinical treatment of OS.

## 4 Ferroptosis in other musculoskeletal tumors

Except for OS, ferroptosis has also been found to inhibit the progression of other musculoskeletal tumors, including Rhabdomyosarcoma and Fibrosarcoma (**Table 2**). Although there are few researches in this field, its importance cannot be ignored. Recognizing the mechanisms of ferroptosis in other musculoskeletal tumors would help gain a deeper understanding of the relationship between ferroptosis and OS and may provide new ideas for further research.

**Table 2:** Ferroptosis-related in other types of musculoskeletal tumor.

Tumor type	Compound	Mechanism	Effects	Reference
Rhabdomyosarcoma	Fenretinide	Accumulating ROS	Inducing ferroptosis	(76)
	Ferrostatin-1	Depleting ROS	Inhibiting ferroptosis	(77)
Fibrosarcoma	HO-1	Accumulating Fe2+	Inducing ferroptosis	(78)
	Serine hydrolase inhibitor	Accumulating ROS	Inducing ferroptosis	(79)
	LOX15 activator	Accelerating lipid peroxidation	Inducing ferroptosis	(80)
	IDH2	Accumulating GSH	Inhibiting ferroptosis	(81)
	Lysosome inhibitor	Depleting ROS	Inhibiting ferroptosis	(27)

Several studies have shown that Erastin and RSL3 can induce ferroptosis in rhabdomyosarcoma and fibrosarcoma cells (77, 82, 83). In contrast, Ferrostatin-1 effectively inhibited the pro-ferroptosis effects of Erastin and RSL3 (77). This is consistent with the findings in OS. Notably, Fenretinide, a synthetic derivative of all-trans-retinoic acid, was found to promote ferroptosis in rhabdomyosarcoma cells by inducing a large accumulation of ROS (76). The Heme Oxygenase 1 (HO-1), Serine hydrolase inhibitor, and LOX15 activator induced ferroptosis in fibrosarcoma cells respectively by causing intracellular iron overload, ROS accumulation, and accelerating lipid peroxidation (78–80). Mitochondrial NADP-dependent isocitrate dehydrogenase (IDH2) and Lysosome inhibitor inhibited ferroptosis in fibrosarcoma cells by accumulating GSH and depleting ROS (27, 81). These findings may provide new targets and drugs for the study of ferroptosis in OS.

## 5 Discussion

As a newly discovered form of PCD, ferroptosis has complex mechanisms and involves a variety of cellular metabolic processes. By summarizing the existing research, we believe that promoting ferroptosis may be an effective method to effectively inhibit tumor progression and reduce chemotherapy resistance, which undoubtedly provides valuable help for solving the key and difficult problems in current tumor treatment.

Focusing on OS, we found that a variety of drugs and biologically active small molecules could inhibit the progression of OS in a ferroptosis-inducing manner, while the discovery of clinically used drugs, such as sulfasalazine and artemisinin, may provide safe drug options for subsequent clinical trials and accelerate the process of clinical application of ferroptosis. In addition, ferroptosis may help reduce chemotherapy resistance in OS. Regarding lung metastasis, it has been reported that ferroptosis has a positive effect on inhibiting the progression of various types of lung cancer and improving prognosis (84), but only one animal experiment has proved that ferroptosis can effectively inhibit the lung metastasis of OS (54). The mechanism and effect of ferroptosis in improving lung metastasis of OS still need further research. Due to the iron-dependent characteristics of ferroptosis, the construction of drug delivery vehicles with high iron content may further enhance the efficacy of carrying chemotherapeutic drugs based on inducing ferroptosis. While compiling these exciting results, we have also identified some limitations of the current research. It mainly includes two aspects: 1. The mechanism of ferroptosis regulation in OS mainly focuses on the GSH-GPX4 axis, and rarely involves the iron metabolism pathway and lipid peroxidation pathway. 2. In addition to chemotherapy, ferroptosis has been found to be closely associated with enhancing the effects of radiotherapy and immunotherapy for tumors (85). However, a large number of current studies have focused on the application of ferroptosis in chemotherapy, ignoring the potential of ferroptosis in non-drug treatments.

Therefore, in the future, continuously improving the mechanisms of ferroptosis, on this basis, finding therapeutically meaningful targets, and developing related targeted drugs is the main research direction of ferroptosis in the field of OS. In addition, it is also meant to explore its application value in the field of non-drug therapy.

## Author contributions

KY conceived and critically revised the manuscript. XL, SD, and SW performed the literature search and data analysis. XL was the major contributor to the drafting of the manuscript. All authors contributed to the article and approved the submitted version.

## Acknowledgments

The authors would like to thank all the reviewers who participated in the review.

## Conflict of interest

The authors declare that the research was conducted in the absence of any commercial or financial relationships that could be construed as a potential conflict of interest.

## Publisher’s note

All claims expressed in this article are solely those of the authors and do not necessarily represent those of their affiliated organizations, or those of the publisher, the editors and the reviewers. Any product that may be evaluated in this article, or claim that may be made by its manufacturer, is not guaranteed or endorsed by the publisher.
